# An Overview of VPAC Receptors in Rheumatoid Arthritis: Biological Role and Clinical Significance

**DOI:** 10.3389/fendo.2019.00729

**Published:** 2019-10-22

**Authors:** Rosa P. Gomariz, Yasmina Juarranz, Mar Carrión, Selene Pérez-García, Raúl Villanueva-Romero, Isidoro González-Álvaro, Irene Gutiérrez-Cañas, Amalia Lamana, Carmen Martínez

**Affiliations:** ^1^Departamento de Biología Celular, Facultad de Biología y Facultad de Medicina, Universidad Complutense de Madrid, Madrid, Spain; ^2^Servicio de Reumatología, Hospital Universitario de la Princesa, Instituto de Investigación Sanitaria la Princesa (IIS-IP), Madrid, Spain

**Keywords:** VPAC receptors, vasoactive intestinal peptide, rheumatoid arthritis, inflammation, autoimmunity, prognosis biomarker

## Abstract

The axis comprised by the Vasoactive Intestinal Peptide (VIP) and its G protein-coupled receptors (GPCRs), VPAC1, and VPAC2, belong to the B1 family and signal through Gs or Gq proteins. VPAC receptors seem to preferentially interact with Gs in inflammatory cells, rather than Gq, thereby stimulating adenylate cyclase activity. cAMP is able to trigger various downstream pathways, mainly the canonical PKA pathway and the non-canonical cAMP-activated guanine nucleotide exchange factor (EPAC) pathway. Classically, the presence of VPACs has been confined to the plasma membrane; however, VPAC1 location has been described in the nuclear membrane in several cell types such as activated Th cells, where they are also functional. VPAC receptor signaling modulates a number of biological processes by tipping the balance of inflammatory mediators in macrophages and other innate immune cells, modifying the expression of TLRs, and inhibiting MMPs and the expression of adhesion molecules. Receptor signaling also downregulates coagulation factors and acute-phase proteins, promotes Th2 over Th1, stimulates Treg abundance, and finally inhibits a pathogenic Th17 profile. Thus, the VIP axis signaling regulates both the innate and adaptive immune responses in several inflammatory/autoimmune diseases. Rheumatoid arthritis (RA) is a complex autoimmune disease that develops on a substrate of genetically susceptible individuals and under the influence of environmental factors, as well as epigenetic mechanisms. It is a heterogeneous disease with different pathogenic mechanisms and variable clinical forms between patients with the same diagnosis. The knowledge of VIP signaling generated in both animal models and human *ex vivo* studies can potentially be translated to clinical reality. Most recently, the beneficial effects of nanoparticles of VIP self-associated with sterically stabilized micelles have been reported in a murine model of RA. Another novel research area is beginning to define the receptors as biomarkers in RA, with their expression levels shown to be associated with the activity of the disease and patients-reported impairment. Therefore, VPAC expression together VIP genetic variants could allow patients to be stratified at the beginning of the disease with the purpose of guiding personalized treatment decisions.

## Introduction

Rheumatoid arthritis (RA) is a chronic autoimmune disease that causes joint inflammation and cartilage and bone destruction. RA affects 1% of the population worldwide and it is associated with damage of physical condition and quality of life. RA progresses in genetically susceptible individuals, under the effect of environmental factors, as well as with the contribution of epigenetic mechanisms. Vasoactive Intestinal Peptide (VIP) and its G protein-coupled receptors (GPCRs), VPAC1, and VPAC2, shape an axis signaling that regulates both the innate and immune response in several inflammatory/autoimmune diseases.

In the following sections, we will describe its therapeutic role in animal models of RA and its potential as a biomarker for the stratification of patients, contributing to the improvement of the development of personalized therapies.

## VPAC Receptors: Structure, Signal Transduction, Cellular Localization, Agonist/Antagonist Molecules, and Detection

VIP is a broadly distributed neuropeptide with known immunomodulatory attributes. VIP can act through three receptors: VIP acts almost exclusively through two high affinity receptors: VPAC1 and VPAC2. A third receptor, PAC1, poorly binds VIP in most physiological contexts, acting primarily by binding to the related ligand PACAP. In RA, VIP expression has been reported in nerve endings and in other cell types in the inflamed area, and it has been shown to actively modulate immune responses in experimental arthritis. In this regard, VIP released from sensory axonal terminals interacts with other molecules produced by nearby neutrophils, macrophages, and other immune and endothelial cells (1). Lymphocytes and synovial fibroblasts (SF) also produced and released VIP (2–5).VPAC1 and VPAC2 belong to the B1 family of GPCRs. PAC1, along with the secretin receptor, the glucagon receptor, the parathyroid hormone receptors and several others, are also found in this family. The members of this GPCR family share some specific characteristics (6, 7): (i) the presence of a large N-terminal ectodomain containing six highly conserved cysteine residues connected by three disulfide bridges; (ii) the N-terminal acting as the major binding site for its natural peptide ligand; (iii) the existence of a signal peptide; (iv) the presence of N-glycosylation sites; (v) the lack of archetypical family A GPCR motifs such as E/D-R-Y or NP-xx(x)-Y; and (vi) the presence of many introns in their gene organization. This last point indicates that alternative splicing events could arise within VPAC receptors, however, although there is some evidence for the presence of splice variants of the VPAC1 and VPAC2 receptors, their functional significance is not yet clear (8). Studies with CHO cell line indicate that these receptors can also form oligomeric complexes, like other GRPRs, VPAC1 receptor is able to homodimerize and heterodimerize with VPAC2 or the secretin receptor (9). This oligomerization does not affect the recognition of ligands nor the function of the receptors (10).

It has been proposed that the interaction between natural ligands and VPAC receptors follows the “two-site” model, in the same way as in other members of the B1 family (6, 7). In this model, the central and C-terminal parts of the peptide ligand are trapped by the N-terminal of the receptor, which ensures correct ligand positioning. Binding of residues 1–6 of the ligand to the extracellular loops and transmembrane helices leads to receptor activation. To determine this, most studies have used VIP as the natural ligand and VPAC1 as the receptor (11, 12).

**Figure 1:**
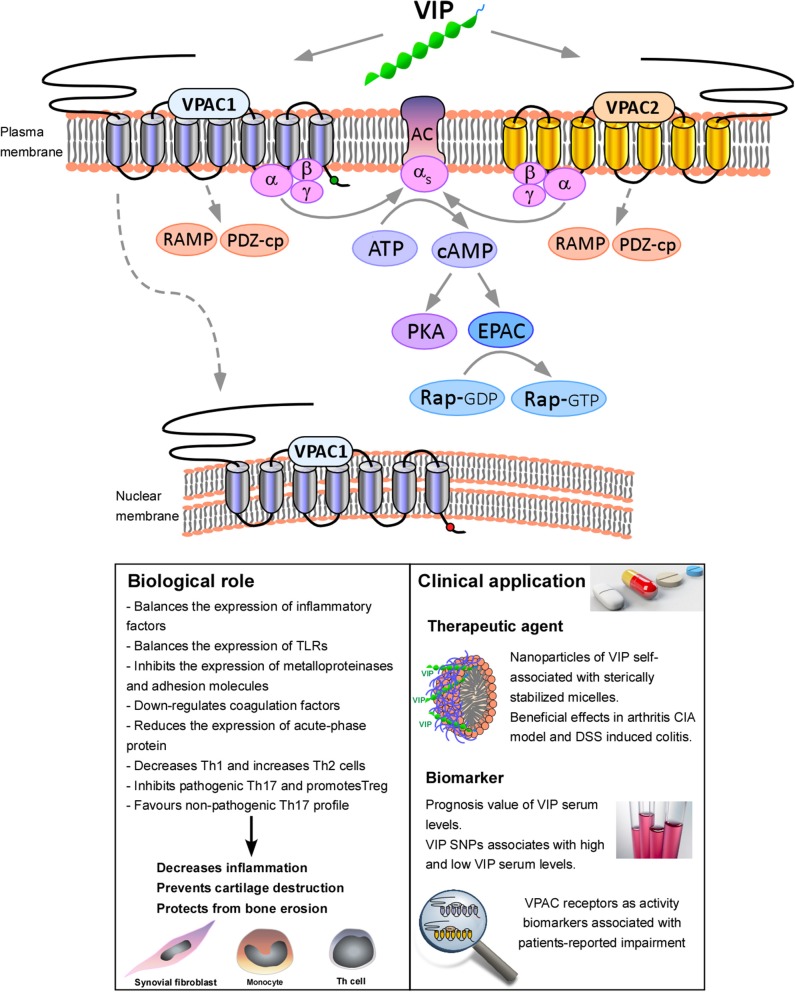
Functional consequences of VPAC receptors signaling. VIP binds both, VPAC1 and VPAC2. VPAC receptors have greater affinity for Gαs than Gαq, indicating that VPAC receptors preferentially stimulate adenylyl cyclase (AC) activity, increasing cAMP levels and triggering downstream pathways, mainly the canonical PKA pathway and the non-canonical cAMP-activated guanine nucleotide exchange factor (EPAC) pathway. Moreover, these receptors can also interact with accessory GPCR interacting proteins (GIPs), such as RAMPs or PDZ-containing proteins (PDZ-cp). In addition, VPAC1 is also able to translocate to nucleus. Activation of VPAC receptors signaling modulates a number of biological processes, which eventually lead to the decrease of the inflammation, cartilage destruction and bone erosion. In relation to the clinical application, it has been reported the beneficial effects of nanoparticles of VIP self-associated with sterically stabilized micelles (SSMs) in collagen-induced arthritis (CIA) murine model (18, 19) and DSS induced colitis (20). We have also demonstrated the prognosis value of VIP serum levels and VIP genetic variants. Moreover, VPAC receptors expression has been described as activity biomarkers associated with patients-reported impairment (21–23).

VPAC receptors signal through Gs or Gq proteins, class B receptors in general demonstrate greater potency in cAMP than IP3 generation, but this depends on cell type. In inflammatory cells, VPAC have greater affinity for Gαs than Gαq, indicating that VPAC receptors preferentially stimulate adenylyl cyclase activity. In addition, it has been demonstrated in cell lines that these receptors can also interact with non-G proteins, which are termed “accessory proteins” or “GPCR interacting proteins (GIPs),” such as RAMPs or PDZ-containing proteins (6, 7, 13, 14). This interaction can modulate several properties of VPAC receptors including their ability to interact with G proteins or their agonist-induced internalization. As mentioned above, adenylyl cyclase activation and the subsequent increase in cAMP levels is the main signaling pathway of VPAC receptors. However, cAMP is able to trigger various downstream pathways, mainly the canonical PKA pathway and the non-canonical cAMP-activated guanine nucleotide exchange factor (EPAC) pathway. Depending on the cell type, EPAC and PKA may act independently, synergistically, or may oppose each other regulating specific cellular functions (15). In addition to the PKA-dependent pathway, it has been shown that VPAC receptors can also activate the PKA-independent pathway in several cell types (16, 17). On the other hand, binding of Gαq to VPAC is involved in the activation of the phospholipase C (PLC) pathway, stimulating protein kinase C (PKC) and promoting release of Ca^2+^ from the endoplasmic reticulum [(8, 10); Figure 1].

VPAC receptors, as with other GPCRs, are synthesized by the protein secretory pathway and their expression and trafficking are under co- and post-translational quality control to ensure that only proper VPAC receptors reach the cell surface (24). These receptors can also undergo internalization by homologous or heterologous desensitization (25, 26). In regards to the VPAC1 receptor, the internalization from plasma membrane by endosomes is induced by its own ligand, VIP. In addition to the plasma or endosome membranes, the intracellular position of functional GPCRs now broadly accepted by the scientific community (27, 28). The presence of VPAC1 receptor has been described in intracellular compartments, such as the nuclear membrane in different cells, as human breast cell lines, glioma cell lines or activated *ex vivo* Th lymphocytes, where they are also functional (17, 29–31). In this sense, the palmitoylation of the N-terminal extracellular Cys37 of the VPAC1 receptor induced by VIP mediates the nuclear translocation of this receptor (31). The trafficking of the VPAC1 receptor from the plasma membrane to the nuclear membrane could be related to the fact that the peptide sequence of this receptor has a nuclear localization signal sequence in its intracytoplasmic C-terminal, not found in the VPAC2 receptor (30). In this sense, the presence of VPAC2 in intracellular locations other than endosomes has not yet been described.

The development of selective agonists or antagonists for GPCR is essential for studying these receptors, both from research and pharmacological points of view. These molecules must possess ligand-selectivity, cross-species reactivity and receptor subtype selectivity. Different VPAC receptor agonists and antagonists have been developed over the years, however, only a few of them have all these characteristics and are broadly recognized by the scientific community. Thus far, there are two selective agonists for the VPAC1 receptor, [Ala^11, 22, 28^]VIP (32) and [Lys^15^, Arg^16^, Leu^27^]VIP (1–7)/CRF (8–17, 24–33). A molecule derived from this agonist, AcHis^1^ [DPhe^2^, Lys^15^, Arg^16^, Leu^27^]VIP (1–7)/CRF (8–17, 24–33), also called PG97-269, is currently the only selective antagonist for the VPAC1 receptor (34). Ro 25-1553 and Ro 25-1392 are the only selective agonists for the VPAC2 receptor described thus far (35, 36). An artificial VPAC2 receptor antagonist, the cyclic peptide VIPpep-3 (Ac-CPPYLPRLCTLLLRS-OH), which has the characteristics abovementioned for a good antagonist, has recently been described (37). Studies with VPAC1 and VPAC2 chimeric receptors have shown that different agonists or selective antagonists of these receptors require interactions with different domains in the receptors to generate their selectivity (38). For example, the N-terminal extracellular domain is responsible for the selectivity of the PG97-269 VPAC1 antagonist, whereas the selective recognition of the VPAC1 agonist, [Lys^15^, Arg^16^, Leu^27^]VIP (1–7)/CRF (8–17, 24–33), is supported by a large receptor area, namely the N-terminal domain, the first extracellular loop, and additional determinants in the distal part of this receptor (39). Moreover, new delivery strategies are being developed to improve the potency, selectivity and pharmacokinetics of VIP. The most striking development is nanotechnology, which provides a VIP-controlled drug delivery, avoiding the peptide breakdown by proteases and digestive acids before it reaches its target (40).

In light of this, we could consider that the traditional point of view, in which cell surface GPCRs are the only targets for therapeutic beneficial drugs or for studying their signaling pathways and functional consequences, is incomplete. Upon activation at the cell surface, many GPCRs with their agonists traffic through intracellular compartments like endosomes or the nucleus, where they can continue to signal. VPAC receptors are no different from other GPCRs, and thus, it is important to define what functions are exerted by these receptors, depending on their location in the cell. It is necessary to develop better techniques to reveal the molecular details of signaling by unmodified VPAC receptors and their spectrum of signaling and regulatory partners in subcellular microdomains of functionally relevant cells in real time (41). Approaches such as cryoelectron tomography, which can provide structural information about protein signaling complexes in intact cells, or GPCR ascorbate peroxidase (GPCR-APEX), which can be used for the identification of proteins that are closely associated with GPCRs by mass spectrometry, will be very useful (41). In addition, it is necessary to develop VPAC receptor agonists or antagonists that specifically target intracellular VPAC receptors to achieve more specific modulation of their physiological responses.

## VIP/VPAC Axis in Animal Models of Rheumatoid Arthritis

Collagen-induced arthritis (CIA) is a model that can be induced in mice, rats or primates. It shares different features with RA, as immunization of animals with type II collagen leads to a chronic, destructive polyarthritis (42) and thus, this model is widely used to study the mechanisms involved in this disease. In 2001, Delgado and colleagues described for the first time how VIP treatment of CIA mice improves the progression of the disease, reducing the incidence and severity of arthritis and avoiding joint swelling and destruction of cartilage and bone (43). Mice treated with VIP daily were best protected from the disease, although a single administration at the onset of CIA was enough to decrease the severity. The mechanisms underlying the improvement of the disease are immunomodulatory as VIP regulates T helper (Th1/Th2 balance, diminishing Th1 and augmenting Th2 cytokines and cells, and it decreases the T helper: T suppressor ratio. Moreover, VIP also affects the inflammatory components of the disease, as VIP treatment downregulates numerous pro-inflammatory factors, as well as several chemokines while it increases anti-inflammatory cytokines. Furthermore, VIP also affects the inflammatory components of the disease, as VIP treatment downregulates numerous pro-inflammatory factors, as well as several chemokines while it increases anti-inflammatory cytokines. Moreover, VIP decreases the expression and activity of matrix metalloproteinases, therefore inhibiting cartilage destruction and bone erosion. The main receptor involved in VIP effects on arthritis is VPAC1, as demonstrated by the use of specific agonists in arthritic mice (43). VIP inhibits the LPS-induced release of cytokines involved in the production of pro-inflammatory mediators in CIA rats, and reduces the TNFα-induced proliferation of synovial cells by downregulating the enhanced expression and activity of NFκB transcription factor. Moreover, the use of a VPAC antagonist counteracts the effect of VIP (44).

Some studies have corroborated the anti-inflammatory role of VIP in the CIA mice model, deepening our understanding of the pathways, receptors, and cell types involved. For instance, VIP is able to diminish the expression of inflammatory mediators and cytokines related to bone destruction, while enhancing the expression of those related to bone protection by means of NFκB and Jun N-terminal kinase pathways (45). Moreover, a selective VPAC1 agonist is able to reduce the chronic inflammation of synovial tissue, the expression of inflammatory cytokines and chemokines, cartilage destruction, and bone erosion, whereas the selective VPAC2 agonist is not (46). Synovial cells are the major producers of innate immune inflammatory mediators, but dendritic cells (DCs) are also able to produce these mediators, driving, at least in part, inflammation in RA. In this regard, a recent study showed that DCs differentiated from monocytes in the presence of VIP are tolerogenic DCs which, when administrated to CIA mice, reduce the arthritic scores (47).

Studies in the CIA rat model demonstrate that VIP reduces the severity of the disease by regulating the balance of Th1/Th2, enhancing regulatory T cells (Treg) and reducing Th17 cells, modulating the mediators of bone erosion (48, 49). Moreover, the expression of the three VIP receptors on osteoclasts is downregulated with the onset of the disease in this model, and VIP diminishes the activity of these bone-related cells (50). VPAC1 expression is shown to be downregulated in synovial tissue of CIA rats and is enhanced by electroacupuncture treatment while VPAC2 remains the same (51). In the most recent paper published thus far, the authors hypothesized that VIP downregulates the CIA rat model by virtue of the enrichment of regulatory monocytes (52).

Given the short half-life of this peptide, some attempts have been made to achieve better targeting to inflammation sites of VIP, with lower doses required, such as viral and non-viral vectors to deliver VIP by gene therapy, the generation of latent forms of this peptide by fusion to latency-associated peptides, or the administration of VIP inside stabilized micelles. All of these innovative forms of delivery have been assayed in the CIA model of rheumatoid arthritis (18, 53, 54).

Other models that have been very useful in the study of the VIP/VPAC axis are the genetically modified mice, knockout mice, which have served to better understand the involvement of endogenously produced VIP. Mice lacking VIP gene by homologous recombination, were able to survive to weaning, despite the wide distribution of this peptide in the organism, but with important alterations in circadian rhythms (55). Moreover, VIP KO mice exhibit changes in reproductive function, behavior, feeding, metabolism, bladder function and gastrointestinal tract as it has been recently reviewed (56). PACAP, VPAC1, VPAC2, and PAC1 knockout mice have also been developed which has served to identify the involvement of these receptors in different physiological processes (56). All these KO models have shown normal basal immune characteristics, although when subjected to immune diseases models, they exhibited impaired immune responses. For instance, VIP KO developed more severe dinitrobenzene sulfonic acid (DNBS)-induced colitis, exacerbated inflammatory asthma and enhancement of innate and adaptive immune responses in a model of viral infection than wild-type mice. However, opposing effects in several inflammatory/autoimmune models developed in VIP KO mice such as LPS-induced endotoxemia, TNBS- and DSS induced-colitis and experimental autoimmune encephalomyelitis (EAE), a model for multiple sclerosis, have been reported (57–59). To date, there is no clear explanation for these results and different hypotheses have emerged that should be verified. Among others, it has been proposed that under some conditions the involvement of VIP could be essential for the whole expansion of immune responses. The existence of compensatory mechanisms by PACAP and the involvement of microbiota have also been suggested (56). VPAC1 KO mice showed diminished inflammatory responses in DNBS-induced colitis and a resistant phenotype to EAE. Besides, VPAC2 KO mice were subjected to skin immediate-type (ITH) and delayed-type (DTH) hypersensitivity models and showed enhanced DTH with augmented Th1 but reduced Th2 cytokine generation and reduced ITH, suggesting a protective role of this receptor against Th1-driven diseases, furthermore, VPAC2 KO mice exhibited exacerbated EAE compared to wild-type mice (56). Unfortunately, as far as we know, there are no studies carried out with the CIA model in mice deficient of VIP, which could serve to corroborate the data described above.

## VPAC Receptors Signaling Control Immune Response in Healthy and RA Patients

Immune dysregulation is at the core of RA pathogenesis, implying disordered immune responses at both systemic and local levels. Furthermore, joints affected by RA characteristically exhibit an altered hyper-activation state of the synovial stromal tissue, which also plays a critical role in the joint pathology. During RA progression, the synovial lining layer expands up to 10–12 cells in thickness, comprised of both SF and macrophages, and the sublining is infiltrated by adaptive and innate immune cells, including B and T lymphocytes as well as inflammatory monocytes, which will differentiate into macrophages (60).

### Synovial Fibroblasts

Joint resident RA synovial fibroblasts (RASF) are mesenchymal stromal cells with immunomodulatory capabilities that secrete cytokines, chemokines and matrix damaging enzymes, actively contributing to synovitis, and ultimately, to cartilage/bone destruction (61, 62). RASF assume an autonomous pathogenic phenotype characterized by the capacity for hyperproliferation and migration, which contributes to the synovium hyperplasia and to the spread of the disease to unaffected joints (63). The pathogenic behavior of RASF is activated and enhanced in response to multiple pathways, including pro-inflammatory mediators and Toll-like receptor (TLR) ligands present in the RA synovium (64).

VIP anti-inflammatory signaling is functional in RASF, decreasing the production of pro-inflammatory cytokines and chemokines (65), also showing immunomodulatory effects on TLR2 and TLR4 through a negative regulation of their expression and function (66). Moreover, VIP exerts an inhibitory action over several RNA sensors involved in the activation of RASF, including TLR3, TLR7 and helicases RIG-I and MDA-5 (67). VIP is also able to counterbalance the enhancing effect of pro-inflammatory molecules on the expression of IL-17 receptors by RASF and on their production of IL-12 and IL-23 cytokines, which are involved in the facilitation of Th1 and Th17 differentiation, respectively (68).

Effects of VIP on RASF are mediated by its receptors, VPAC1 and VPAC2, which exhibit a differential expression pattern when compared to osteoarthritis (OA) SF. VPAC2 is the dominant receptor in RASF whereas VPAC1 is preferentially detected in OASF, with no presence of PAC1 receptor in either type of SF (4, 69). It is worth noting that TNFα-treated OASF adopt a pattern of expression for VPAC receptors equivalent to RA, suggesting a modulatory effect of the pro-inflammatory synovial milieu on the VPAC system. The functional activity of VPAC receptors in OA and RASF is coupled to adenylate cyclase (AC), and specific agonists for each receptor subtype are equivalent to VIP in inducing an increase in intracellular cAMP levels by activating the dominant receptor, VPAC1 and VPAC2, respectively. Specifically, the VPAC2-specifc agonist reproduces the inhibitory effects of VIP on pro-inflammatory CXCL8, IL-6 and CCL2 production by RASF (4). Therefore, VPAC2 is the receptor mediating the immunomodulatory effects of VIP on RASF through the activation of the AC pathway.

### Monocytes/Macrophages

RA synovial macrophages are considered central effectors in the progression of joint degeneration, by secreting several pro-inflammatory cytokines and joint-damaging mediators (70). In fact, pathological contribution of SF to RA progression is directly linked to both resident and monocyte-derived infiltrating macrophages (71, 72). The number of synovial sublining macrophages is correlated with the degree of joint erosion and disease activity in RA patients (73). Specifically, RA synovial macrophages exhibit a transcriptomic and protein profile that is similar to that of macrophages polarized *in vitro* by GM-CSF (GM-MØ), also known as M1. GM-MØ are characterized by the expression and secretion of pro-inflammatory factors in contrast to macrophages polarized by M-CSF (M-MØ), also known as M2, that display an anti-inflammatory profile (74).

Most studies demonstrate that VPAC1 is constitutively expressed at higher levels than VPAC2 in resting human monocytes/macrophages, whereas VPAC2 expression is induced by immune stimulation (75–78). Other authors have described the expression of PAC1 and the absence of VPAC2 in these cells, probably as a result of the different methods used (77–79). More recently, the analysis of VPAC receptors expression during the time course of the *in vitro* polarization of monocytes toward GM-MØ or M-MØ showed a progressive down-regulation of transcripts for VPAC1 and VPAC2, detecting higher levels for both receptors in GM-MØ at the end of the process. Likewise, RA synovial macrophages, according to their GM-CSF-like polarization state, exhibited a higher expression of VPAC1 and VPAC2 compared to macrophages from a non-inflamed synovium (78).

Regarding the functional activity of VPAC receptors in human monocytes, inhibitory signals of the VPAC2-specific agonist on LPS-induced synthesis of TNFα and IL-12 have been described (75). Likewise, VIP is able to reduce the synthesis of TNFα, IL-6, and IL-12 by *in vitro*-generated GM-MØ stimulated with LPS. In this regard, specific agonists for VPAC receptors induce an increase in intracellular cAMP levels in GM-MØ, with the VPAC1 agonist reaching levels comparable to those induced by VIP. Moreover, the presence of either VPAC1 or VPAC2 agonists during the GM-CSF-driven *in vitro* polarization impairs the expression of GM-MØ-specific gene markers and up-regulates some genes linked to M-MØ, changing the macrophage profile to a less-damaging phenotype (78).

### Helper T Cells (Th) Cells

Naïve CD4+ Th cells can differentiate into several effector subsets called Th1, Th2, Th17, Th9, Th22 or follicular helper T (Tfh), and Treg. The differentiation of these subsets is coordinated by complex regulatory networks that allow for shared transcriptional programs and plasticity across T cell subsets (80, 81). Effector Th cells (Th1, Th2, Th17, Th9, Th22, Tfh) are important for protective immunity and Treg are responsible for the tolerance of the immune system, however some of these subsets have been related to a greater or lesser extent with malfunctions of the immune system, specifically autoimmune diseases, like RA (82, 83). Before the discovery of Th17, RA was thought to be due to an increase in the Th1 subset and a loss of tolerance, Treg. Nowadays, Th17 cells have acquired a key role in the pathogenesis of RA (82–85). This subset is heterogenic and can show a pathogenic or non-pathogenic profile, depending on the cytokine balance present in the microenvironment during its differentiation/activation. In addition, Th17 cells are the most plastic subset of all the others. Pathogenic Th17 cells are directly involved in RA and are able to change their lineage commitment, shifting to Th1 cells (termed “non-classical Th1” cells or “Th17/1” cells), which are reported to be more pathogenic than Th17 cells per se in RA (84–87).

As stated above, in the animal model of RA, the CIA model, VIP is able to rebalance Th1/Th2 subsets in the immune system, decreasing Th1 cells and increasing Th2 cells, and to downregulate Th17 responses. Furthermore, VIP also expands Treg cells in the periphery in this animal model, which inhibits autoreactive T cell activation/expansion, for example increasing Treg/Th17 balance (43, 48, 88, 89). In peripheral blood lymphocytes of RA patients cultured *ex vivo*, VIP favors the Th2/Treg profile (90). In addition, VIP reduces the Th17 and Th17/1 pathogen profile of memory Th cells of early RA patients activated/expanded *ex vivo* (89, 91). Even when memory Th cells from early RA patients are polarized *in vitro* to a non-pathogenic Th17 profile in the presence of TGFβ, they maintain a pathogenic profile (92). However, the presence of VIP during this Th17 polarization, increases their Treg/Th17 profile, characteristic of non-pathogenic Th17 cells, and decreases the Th17/Th1 profile, characteristic of the pathogenic phenotype (89, 92).

Th cell activation induces changes in the expression of VPAC receptors and their cellular location (17, 79, 86, 89, 91–93). Th lymphocytes from mouse and human express both VPAC1 and VPAC2 receptors and their mRNA expression patterns change between Th cells from mouse, healthy donors and patients with early RA. Activation of Th cells results in a loss of VPAC1 mRNA expression, the range of expression of VPAC1 is between 11- and 61-fold higher in resting Th cells than activated cells, however no changes at the protein level are detected in Th cells from healthy donors or from early RA patients (17, 79, 89, 91). The VPAC2 mRNA and protein expression is up-regulated after *in vitro* activation, whereas low levels of VPAC2 are present in resting Th cells, the range of expression is between 2- and 5-fold higher in activated Th cells than resting cells (17, 79, 89). In relation to their cellular location changes, in resting Th cells, VPAC1 is found both on the cell surface and intracellularly, whereas when these cells are activated, this receptor is found exclusively inside the cell. This change in the receptor cellular location is not observed for VPAC2 since it is always on the cellular surface, regardless of the activation state of Th cells (17). As early as Goetzl (94) hypothesized that VPAC receptors constitute a dynamic system for signaling in T cells, predicting that responses in the plasma membrane location would have a fast onset and brief duration, whereas receptors in the nuclear membrane would have responses with slow onset, sustained in time (94). This fact is reflected in the signaling pathways that these receptors trigger, as they signal through a PKA-dependent pathway in resting or activated Th cells, and also by a PKA-independent pathway in activated cells (17). In this way, both receptors exhibit a potent immunomodulatory capacity by controlling the pathogenic profile and the activation markers of Th cells.

As it is above mentioned, the implication of Th1 cells was initially ascribed to RA, however the discovery of the Th17 subset indicated a central role for these cells in this pathology. VIP is able to modulate the differentiation and polarization of these subsets in cells from RA patients. Thus, changes in the pattern expression of VPAC are also important for the pathology. In this context, the differentiation/polarization of Th cells also induces changes in the expression of VPAC receptors; for example, the differentiation/polarization toward Th17 profile induces an increase in the VPAC2/VPAC1 ratio, for example, 18-fold in Th17-differentiated cells from naïve Th cells (86, 92). During the process of differentiation toward a non-pathogenic Th17 profile, VPAC receptors have shown different roles in regulating the production of inflammatory mediators (86).

When VPAC receptor expression in Th cells from healthy donors is compared with that of patients with early RA, the VPAC2/VPAC1 ratio is 2.8-fold higher in cells from early RA patients (17, 91, 92). These data perfectly correlate with the VPAC receptor expression in PBMCs from early RA patients as we described. Briefly, there is a loss of VPAC1 mRNA expression and an increase in VPAC2 expression in these patients (23). We can conclude that VIP is a beneficial modulator that counterbalances the different Th subsets and underscores the importance of VPAC expression in RA.

## Clinical Significance of VIP and VPAC Receptors

### Could VIP Be Useful as a Drug?

The knowledge generated in animal models and in human *ex vivo* studies related to VIP and its signaling pathways could be translated to clinical reality as two potential tools: as a therapeutic agent and as a biomarker in inflammatory/autoimmune diseases.

Although RA disease has no cure, in the last two decades, rheumatologists through an early diagnosis and rapid initiation of an effective therapy are achieving disease remission or at least significantly reducing the activity of the disease.

The present management strategy of RA, accepted by ACR, EULAR, and the Asia Pacific League of Associations for Rheumatology, is the “treat to target” method, which is based on the strict control of the disease activity and the modification of the treatment if the goal is not achieved (95–97).

The currently used therapeutic strategies include the non-steroidal anti-inflammatory drugs (NSAIDs) as symptomatic agents, glucocorticoids used for prevention of joint destruction, and the disease-modifying anti-rheumatic drugs (DMARDs). DMARDs comprise both synthetic (conventional drugs such as methotrexate and targeted synthetic drugs such as JAK inhibitors) and biological agents designed for the specific inhibition of molecules of the immune system, such as TNF, IL-6, IL-6 receptor, CD80-CD86, and CD20. The best results are being achieved with the combined therapy of biological agents and methotrexate. This approach to RA remission produces an improvement of the physical function and prevention of evolution of joint damage. Nevertheless, many patients fail to decrease disease activity and advances in the development of new or complementary therapies are still required (98).

An efficient translation of preclinical studies to the field of health for the use of VIP as a therapeutic agent faces two important challenges. On the one hand, it has low metabolic stability with a short *in vivo* half-life, due to its degradation by proteases, by spontaneous hydrolysis or by catalytic antibodies (54). On the other hand, the systemic administration of VIP can cause side effects due to the cross-interactions of binding to three GPCRs, its functional pleiotropism and its ubiquity. Severe decreases of the blood pressure, and alterations in the cardiovascular and gastrointestinal system such as the watery diarrhea syndrome (99, 100), have been documented. Thus, VIP-mediated therapeutic intervention in RA management must include the design of distribution systems directed against specific targets and the protection of the peptide against its degradation. Unfortunately, none of the strategies designed, such as the use of VIP analogs or the application of protease inhibitors, have rendered effects for use in systemic therapy.

With this background, the field of nanoparticle engineering has been developed over the last few years, generating attractive data, although not yet sufficient, for clinical application (40). Two strategies have been developed with VIP-based nanoparticles: (a) to act itself as a drug by means of encapsulation or by its attachment to the surface of the nanoparticle; or (b) to serve as a vehicle for another compound to a precise location. In this second strategy, phospholipid nanomicelles spliced with VIP target acting as carriers for water-insoluble anticancer drugs has been used in breast cancer (101). The same approach has been used in RA in a study testing a new indication for the anti-cancer drug camptothecin a Topoisomerase I inhibitor. Camptothecin sterically stabilized micelles conjugated with VIP (CPT-SSM-VIP) has been designed to be delivered in specific cell types, such as synoviocites, T-cells and macrophages, where VPAC2 are over-expressed. This approach of actively targeting long circulating micelles to the effector cells in RA joints improved the efficacy of the drug and reduced systemic toxicity. They reported that a single, subcutaneous injection of low-dose camptothecin preparation alleviated joint inflammation in the CIA model (102).

Regarding the strategy of nanoparticles developed for VIP as a drug, recently the beneficial effects in the CIA murine model have been reported by targeting low doses of VIP self-associated with sterically stabilized micelles (SSMs) [(18, 19); Figure 1]. This spontaneous interaction of VIP with SSM protects the peptide from enzymatic degradation or inactivation in biological fluids and prolongs circulation half-life. The small size of the VIP particles (~15 nm) prevents the nanoparticle from extravasating through undamaged blood vessel walls abolishing vasorelaxation and also carrying the particles to the sites with “permeable” vasculature, such as inflamed tissues. While the authors suggest that the components of VIP-SSM formulation have been used for humans in marketed pharmaceutical products such as InvicorpR and DoxilR, there are still problems to be solved regarding their biodistribution, not only in the joints but also in the kidney and liver (18).

In a recent study, this strategy confirmed that VIP-SSM prevented and ameliorated severe inflammation related to DSS-induced colitis, in a murine model of inflammatory bowel disease. The treatment reversed the augmented pro-inflammatory cytokine expression (IL-1β, CXCL1, CXCL2, and CCL3), injured distal colonic histology, and reduced tight junction (occludin expression) and ion transporter protein expression associated with severe DSS colitis [(20); Figure 1].

The negative side of the current systemic RA treatments, previously described, is the appearance of side effects. These are generated by the toxicity associated with long-term treatments and large doses that these drugs induce. To evade the off-target toxicity of these drugs, a potential solution is the development of joint-targeting drug delivery. The VIP-based strategy for targeted RA still requires more research, and the resolution of different problems, being one of them, to guarantee their arrival exclusively to the joint.

### Tracing the Potential of the VIP/VPAC Receptors Axis as Biomarker

The early recognition and treatment of RA is hampered by its heterogeneous nature. Current strategies for the management of RA focus on identification and intervention in the “window of opportunity,” the initial period of the disease in which joint damage can be avoided through early and continuous therapy to slow or stop the evolution of the disease (21, 103) as discussed above. Thus, new challenges emerge in the search of prognostic biomarkers to guide personalized treatment decisions.

Given these concerns, we have recently described the utility of measuring VIP as a clinical biomarker in patients with early arthritis (EA) (104). We demonstrate an association between VIP serum levels and genetic variants in the VIP gene and describe certain combinations of genotypes of these variants associated with a greater intensity of treatment in our patients (21, 23). These results highlight the relevance of the detection of VIP genetic variants that allows the stratification of patients as a complementary tool for supporting therapeutic decisions.

New evidence underscores the importance of the VIP/receptors axis through the association of expression levels of VPAC receptors with the clinical status of EA patients (22). In a population of early arthritis EA patients, we evaluated the expression of VPAC1 and VPAC2 in PBMCs during the clinical follow-up (22). We reported that the expression of VPAC1 increased significantly reaching the highest values after 2 years of follow-up, whereas VPAC2 expression decreased. Thus, we described an inverse correlation between the expressions of both receptors during the course of EA suggesting a possible dynamic regulation in the different stages of the disease probably as a compensatory mechanism (22).

The assessment of the activity of RA by several variables is of crucial importance in daily clinical practice with the aim of reducing inflammation and achieving remission. Elevated levels of IL-6 in the serum of patients are considered a marker of inflammation correlating directly to the clinical activity of the disease and to joint destruction (105, 106). Interestingly, a negative correlation has been described between the levels of VPAC1 and IL-6, parallel to the positive association between the levels of this cytokine and the VPAC2 receptor (22). This result points to the role of the low level of expression of VPAC1 as a marker of a more intense inflammatory process.

What is truly remarkable, however, is the relationship between VPAC1 levels and the activity of the disease measured by the disease activity score (DAS), DAS 28 index. Thus, when we clustered EA patients into 3 sub-groups considering their DAS28 levels (remission-low, moderate, and high disease activity), we found that patients with moderate/high activity of the disease show the lowest levels of VPAC1, while patients in remission have higher levels of this receptor. In addition, the expression of VPAC1 makes it possible to distinguish groups of patients with different degrees of disease activity that could serve as an indicator of disease activity in RA (22).

In light of the above, the evaluation of the global activity of the disease by the patient has been gaining relevance in the determination of the outcome of RA showing a high predictive value (107, 108) and allowing pathways to improvement, not only in terms of the traditional objectives of decreased activity but also in terms of patient satisfaction (109). In this context, it has been demonstrated that lower levels of VPAC1 expression are associated with patient-reported impairment correlating better than other serum biomarkers of activity used in clinical practice, such as the erythrocyte sedimentation rate [(22); Figure 1].

The American College of Rheumatology/European League against Rheumatism (110), the European League against Rheumatism (111) and the international working group to address the objectives (112), recommend frequent evaluation of disease activity in order to reduce, or better eliminate, inflammation (113). Therefore, the identification of new biological markers, which reflect the underlying pathophysiological processes associated with the clinical activity of RA, are the target of numerous investigations.

In this sense and given the ability of VIP to regulate the intensity of the inflammatory process and the immune response, the expression of VPAC1 in RA, associated with the activity of the disease, would reflect the patient's clinical status and complement other serum biomarkers of activity used, such as ESR and C-reactive protein (CRP) (114) which are biased by factors such as the gender and the disease duration (115).

In summary, we can conclude that the VIP/VPAC axis represents a promising biomarker in RA because it could allow patients to be stratified with the purpose of guiding personalized treatment decisions.

## Concluding Remarks

The experimental data explained above demonstrate the VIP/VPAC receptor axis as an important endogenous mediator involved in anti-inflammatory and autoimmunity responses in RA. In recent years, sufficient information has been generated related to the VIP/VPAC receptor axis to yield an effective translation of these preclinical studies. The research area that seems most advanced for clinical application is its role as a biomarker. Important advances are being made toward obtaining the means to ensure that the determination of biomarkers allows an accurate prognosis of the onset as well as the valuation of severity of RA. According to the information reported, it may be that in this scenario, the VIP/VPAC axis occupies an important place as a biomarker of this disease. However, for its clinical application to become a reality, more research is needed. The involvement of genetic, epigenetic and miRNAs in the expression of VPAC receptors, and validation in several cohorts of patients, among other issues, are important challenges that must be addressed in future research.

## Author Contributions

RG, YJ, CM, MC, IG-C, IG-A and AL wrote the manuscript. SP-G and RV-R performed the figures. RG coordinated the manuscript. SP-G and RV-R coordinated the bibliography and the format of the manuscript.

### Conflict of Interest

The authors declare that the research was conducted in the absence of any commercial or financial relationships that could be construed as a potential conflict of interest.
